# Socioeconomic factors affect treatment delivery for patients with low grade glioma: a Swedish population-based study

**DOI:** 10.1007/s11060-019-03378-7

**Published:** 2019-12-27

**Authors:** Louise Carstam, Isabelle Rydén, Sasha Gulati, Bertil Rydenhag, Roger Henriksson, Øyvind Salvesen, Anja Smits, Asgeir Store Jakola

**Affiliations:** 1 Department of Neurosurgery, Sahlgrenska University Hospital, Blå Stråket 5, 41345 Göteborg, Sweden; 2 Institute of Neuroscience and Physiology, Sahlgrenska Academy, University of Gothenburg, Göteborg, Sweden; 3 Department of Neurology, Sahlgrenska University Hospital, Göteborg, Sweden; 4 Department of Neurosurgery, St. Olavs University Hospital, Trondheim, Norway; 5 Department of Neuromedicine and Movement Science, Norwegian University of Science and Technology, Trondheim, Norway; 6 Department of Radiation Sciences and Oncology, University of Umea, Umeå, Sweden; 7 Department of Public Health and Nursing, Norwegian University of Science and Technology, Trondheim, Norway; 8 Department of Neuroscience, Neurology, Uppsala University, Uppsala, Sweden

**Keywords:** Diffuse low-grade glioma, Social disparities, Equal care, Glioma/surgery, Brain neoplasm, Neurosurgery

## Abstract

**Background:**

Despite aspirations to achieve equality in healthcare we know that socioeconomic differences exist and may affect treatment and patient outcome, also in serious diseases such as cancer. We investigated disparities in neurosurgical care and outcome for patients with low-grade glioma (LGG).

**Methods:**

In this nationwide registry-based study, patients who had undergone surgery for LGG during 2005–2015 were identified (n = 547) through the Swedish Brain Tumor Registry. We linked data to multiple national registries with individual level data on income, education and comorbidity and analyzed the association of disease characteristics, surgical management and outcome, with levels of income, education and sex.

**Results:**

Patients with either low income, low education or female gender showed worse pre-operative performance status. Patients with low income or education also had more comorbidities and those with low education endured longer waiting times for surgery. Median time from radiological imaging to surgery was 51 days (Q1–3 27–191) for patients with low education, compared to 32 days (Q1–3 20–80) for patients with high education (p = 0.006). Differences in waiting time over educational levels remained significant after stratification for age, comorbidity, preoperative performance status, and tumor size. Overall survival was better for patients with high income or high education, but income- and education-related survival differences were not significant after adjustment for age and comorbidity. The type of surgical procedure or complications did not differ over socioeconomic groups or sex.

**Conclusion:**

The neurosurgical care for LGG in Sweden, a society with universal healthcare, displays differences that can be related to socioeconomic factors.

**Electronic supplementary material:**

The online version of this article (10.1007/s11060-019-03378-7) contains supplementary material, which is available to authorized users.

## Introduction

Social disparities may affect cancer care and patient survival [1–5]. The particular type of health-care system in a society, as well as the extent of economic and social inequalities, are factors that likely affect the patterns of care. Such differences are unsurprising in societies where access to medical care is closely tied to economic status. However, social disparities, including social gradients for several types of cancers, have also been reported in societies with universal health coverage [6–8].

For patients with brain tumors in general, and high-grade gliomas in particular, previous studies have revealed differences in care as a result of social factors [9–14]. Patients with low-grade glioma (LGG), who are typically younger adults in the very middle of socioprofessional life, may be less prone to encounter such inequalities, although this has been largely unstudied. Here we investigated the association between socioeconomic factors and neurosurgical treatment and survival for patients with LGG in Sweden, a society with universal health coverage.

## Patients and methods

### Participants

We used multiple national registries to extract data on all adult patients (≥ 18 years) with a first-time diagnosis of supratentorial hemispheric diffuse LGG in Sweden in the period 2005–2015. Included tumors were WHO grade II astrocytoma, oligoastrocytoma or oligodendroglioma according to the 2000 and 2007 WHO classification of brain tumors [15, 16]. The SNOMED codes used were: 94003, 94203, 94113, 94103 for astrocytoma, 93823 for oligoastrocytoma and 94503 for oligodendroglioma (for oligoastrocytoma WHO grade II and oligoastrocytoma grade III the SNOMED code is the same 93823). Molecular tumor data was not available. Patients with radiologically suspected LGG but no histologically verified diagnosis were not included in the present study.

### The Swedish Brain Tumor Registry

The patients were identified through the Swedish Brain Tumor Registry (SBTR), which is a regionally based registry of adult patients diagnosed with brain tumors covering data from 1999 and onwards. The SBTR contains detailed, prospectively collected, information on tumor and patient characteristics. The registration rate (defined as the percentage of diagnoses in the SBTR that corresponds to diagnoses reported to the compulsory National Cancer Registry) is generally high (> 90%) but has varied somewhat over time and between regions. In order to provide representative population-based data, we set a minimum registration rate of 80% as a requirement to be included in the analysis at any given year for each region. For this reason, only data from the period 2012–2013 were used in one (out of six) region, while only data from 2009 to 2015 were used in the case of another region. For the four remaining regions, data inclusion covered the entire period 2005–2015. For further details of the SBTR, see Asklund et al. [17].

### Statistics Sweden

Statistics Sweden is a government agency responsible for coordinating the system for the official and objective statistics for general information, investigation, and research in Sweden (www.scb.se). Using the unique personal identification numbers assigned to all Swedish residents, we were able to extract individual data on educational level and income for the patients in our cohort. Such individual level information is rarely available for research purposes and similar studies usually rely on area-based approximations for these factors [18]. We received data per year, using the factual income during the year prior to diagnosis, while educational level was registered for the year of diagnosis. The registry was accessed on June 26th 2017.

### Socioeconomic variables

Numerous different but often interlinked indicators are used to measure *socioeconomic position* (SEP) in health research. In this study, we used income and education, where especially the latter is suggested to be a particularly rich indicator of both early life circumstances and adult resources, as well as occupational possibilities [19].

1. Patients were stratified into tertiles based on total income from business and employment for the year prior to diagnosis. This categorization was made for each year separately, to avoid a time-dependent bias related to a general increase in income over the study period.
2. Educational level was graded according to the Swedish nomenclature for education (SUN2000, initial version) from one to seven. We divided the level of education into three groups: *lower educational level* meaning pre-high school studies only (SUN2000 grade one to two), *intermediate educational level* referring to any length of high-school studies (SUN2000 grade three to four) and *higher educational level* defined as any tertiary education (SUN2000 grade five through seven). These or corresponding educational milestones constitute a commonly used classification of educational level [19].
3. Finally, sex was considered a relevant social parameter and comparisons between males and females were made.

### Comorbidity

To obtain individual level information on comorbidity, we extracted information from the National Patient Registry (NPR), which is one of several registers governed by the National Board of Health and Welfare (NBHW). The reporting to NPR is mandatory, and we received data concerning inpatient and outpatient visits, including diagnostic and procedural codes in the period 2003–2016 excluding primary health care contacts. The underreporting of contacts in NPR has been estimated to be less than 1% according to the NBHW (www.socialstyrelsen.se). The ICD-10 codes were the basis for calculating the Elixhauser comorbidity index [20, 21]. The conditions removed from the index due to possible association with diagnosis of LGG were: G40; epilepsy, G41; status epilepticus, R56; convulsions, R47; dysphasia/aphasia and C70–72; Malignant tumor in central nervous system. Each patient received a score from 0 to 30 based upon comorbid categories present or not. We report categories as 0, 1, 2, and 3 or more.

### Statistical analyses

All analyses were performed with the SPSS ver. 25.0 or newer software. The statistical significance level was set to p < 0.05. All tests are two-sided. Central tendencies are presented as means and standard deviations, or medians and interquartile ranges if skewed. Categorical data were analyzed with the Pearson Chi-square test. Comparisons of continuous variables between groups were analyzed using analysis of variance when normally distributed or the Kruskal–Wallis test if skewed. Time to event-analyses including overall survival are presented in Kaplan–Meier curves and compared using the log-rank test.

To calculate adjusted hazard ratios for univariable and multivariable effect on mortality and waiting time for surgery, we initially planned to use a Cox regression model. However, assumptions for proportional hazards were not met, which is why stratifications for relevant variables were used to create more homogeneous groups. After initial preplanned analyses on associations of income, educational level, and sex with clinical factors and outcome, additional post hoc analyses were made to assess the impact of comorbidity.

### Ethics statement

This study was approved by the Ethical Review Board in Västra Götaland region (Dnr 702–16).

## Results

To compare outcomes in relation to social determinants, we first analyzed our cohort in relation to possible inherent differences between the groups regarding major factors known to affect survival (such as patient’s age, presence of neurological deficits, histology, size of the tumor, and bilateral growth). No such differences were found for income levels (Table 1) or sex (Supplementary Table 1). However, as shown in Table 2, the group of patients with the lowest education in our cohort was approximately ten years older than the group of patients with higher level of education (mean age 43.2 vs 52.7 years). No other differences with respect to the above-mentioned prognostic factors were seen between the groups.

**Table 1:** Clinico-pathological factors related to surgical treatment for diffuse, low-grade gliomas in relation to income levels of patients

	Income level			p-value
	Lower n = 179	Intermediate n = 186	Higher n = 177	
Age, mean (SD)	47.7 (18.2)	45.6 (14.1)	46.0 (11.6)	0.35
Asymptomatic, n (%)	14 (8.3) N = 169	10 (5.7) N = 176	13 (7.6) N = 171	0.62
Focal deficit	76 (43.9) N = 173	60 (33.0) N = 182	61 (35.3) N = 173	0.08
WHO functional status^a^, n (%)				0.002
0: Fully active	84 (48.6)	110 (60.4)	105 (60.3)	
1: Light work possible	44 (25.4)	52 (28.6)	39 (22.4)	
2: Cares for self	29 (16.8)	17 (9.3)	25 (14.4)	
3: Limited self care	13 (7.5)	2 (1.1)	2 (1.1)	
4: Disabled, confined to bed	3 (1.7) N = 173	1 (0.5) N = 182	3 (1.7) N = 174	
Bilateral or multifocal tumor growth, n (%)	24 (13.4)	18 (9.7) N = 185	19 (10.7)	0.52
Tumor size, n (%)				0.09
< 4 cm	62 (39.5)	61 (37.7)	68 (42.8)	
4–6 cm	58 (36.9)	77 (47.5)	55 (34.6)	
> 6 cm	37 (23.6)	24 (14.8)	36 (22.6)	
	N = 157	N = 162	N = 159	
Days from imaging to surgery median (Q1–3)	40 (20–131) N = 175	35 (21–73) N = 185	34 (20–81) N = 176	0.24
Resection (not only biopsy), n (%)	119 (66.9) N = 178	138 (75.0) N = 184	128 (73.6) N = 174	0.19
Postop re-operation due to complication^b^, n (%)	7 (4.5) N = 157	7 (4.3) N = 163	14 (8.9) N = 157	0.14
Histopathology				0.19
Astrocytoma	92 (51.4)	100 (53.8)	77 (43.5)	
Oligodendroglioma	60 (33.5)	67 (36.0)	70 (39.5)	
Oligoastrocytoma	27 (15.1)	19 (10.2)	30 (16.9)	
Number of comorbidities, n (%)				0.004
0	119 (66.5)	151 (81.2)	141 (79.7)	
1	36 (20.1)	27 (14.5)	28 (15.8)	
2	14 (7.8)	7 (3.8)	6 (3.4)	
3 or more	10 (5.6)	1 (0.5)	1 (0.6)	
	N = 179	N = 186	N = 177	

Where data are missing, the actual N is provided in individual cells

*WHO* World Health Organisation, *postop* postoperatively

^a^The WHO/Eastern Cooperative Oncology Group (ECOG) performance score

^b^Any complication within 30 days postoperatively leading to re-operation (for example parenchymal or extracerebral hemorrhage, infection)

**Table 2:** Clinico-pathological factors related to surgical treatment for diffuse, low-grade gliomas in relation to educational levels of patients

	Educational level			
	Lower n = 73	Intermediate n = 238	Higher n = 191	p-value
Age, years, mean (SD)	52.7 (16.3)	44.2 (14.1)	43.2 (12.5)	< 0.01
Asymptomatic, n (%)	7 (10.0) N = 70	18 (8.1) N = 223	11 (5.9) N = 187	0.49
Focal neurological deficit, n (%)	29 (40.3) N = 72	80 (34.6) N = 231	61 (33.0) N = 185	0.54
Bilateral or multifocal tumor growth, n (%)	11 (15.1)	21 (8.9) N = 237	17 (8.9)	0.26
Tumor size, n (%)				0.18
< 4 cm	26 (43.3)	75 (36.4)	72 (40.9)	
4–6 cm	24 (40.0)	93 (45.1)	60 (34.1)	
> 6 cm	10 (16.7) N = 60	38 (18.4) N = 206	44 (25.0) N = 176	
WHO performance status^a^, n (%)				0.046
0: Fully active	32 (45.7)	130 (56.5)	126 (66.7)	
1: Light work possible	23 (32.9)	63 (27.4)	40 (21.2)	
2: Cares for self	11 (15.7)	32 (13.9)	20 (10.6)	
3: Limited self-care	3 (4.3)	5 (2.2)	1 (0.5)	
4: Disabled, confined to bed	1 (1.4) N = 70	0 (0.0) N = 230	2 (1.1) N = 189	
Days from imaging to surgery, median (Q1–3)	51 (27–191)	39 (21–86) N = 235	32 (20–80) N = 189	0.006
Resection (not only biopsy), n (%)	46 (64.8) N = 71	177 (74.7) N = 237	146 (77.7) N = 188	0.11
Postop re-operation due to complication^b^	5 (7.6) N = 66	6 (2.9) N = 209	13 (7.7) N = 160	0.08
Histopathology				0.52
Astrocytoma	34 (46.6)	125 (52.5)	85 (44.5)	
Oligodendroglioma	28 (38.3)	84 (35.3)	75 (39.3)	
Oligoastrocytoma	11 (15.1)	29 (12.2)	31 (16.2)	
Number of comorbidities, n (%)				0.001
0	43 (58.9)	186 (78.2)	164 (85.9)	
1	21 (28.8)	37 (15.5)	22 (11.5)	
2	6 (8.2)	10 (4.2)	4 (2.1)	
3 or more	3 (4.1)	5 (2.1)	1 (0.5)	

Where data are missing, the actual N is provided in individual cells

*WHO* World Health Organisation, *postop* postoperatively

^a^The WHO/Eastern Cooperative Oncology Group (ECOG) performance score

^b^Any complication within 30 days postoperatively leading to re-operation (for example parenchymal or extracerebral hemorrhage, infection)

However, post-hoc analyses on comorbidity showed greater numbers of comorbidities in the lowest income group (Table 1) as well as in the lowest educational group (Table 2), compared to the higher income and educational groups. This is exemplified by the fact that 41% of the patients in the lowest educational group had at least one comorbidity, compared to 14% in the highest educational group (p = 0.001, Table 2). No significant differences were found for comorbidities between males and females (Supplementary Table 1).

### Income level

Data on income level were available from 542 out of the 547 LGG patients. As shown in Table 1, patients in the lowest income group presented with worse pre-operative functional status compared to patients in the higher income groups. There were no significant differences between the groups regarding the waiting time for surgery, type of surgical procedure performed or number of complications leading to re-operation in relation to surgery. The post-hoc analyses on comorbidity revealed that more patients in the lowest income tertile had at least one comorbidity according to the Elixhauser comorbidity index (33.5%, N = 179) as compared to those in the highest income tertile (20.3%, N = 177, Table 1).

### Educational level

Information on educational level for 501 out of the 547 patients was available. We divided the patients into three commonly used categories of educational level, as outlined above. However, as seen in Table 2, this categorization yielded an uneven distribution between groups, with markedly fewer patients in the lowest educational level group (lower education N = 73, intermediate education N = 238, higher education N = 191).

**Fig. 1:**
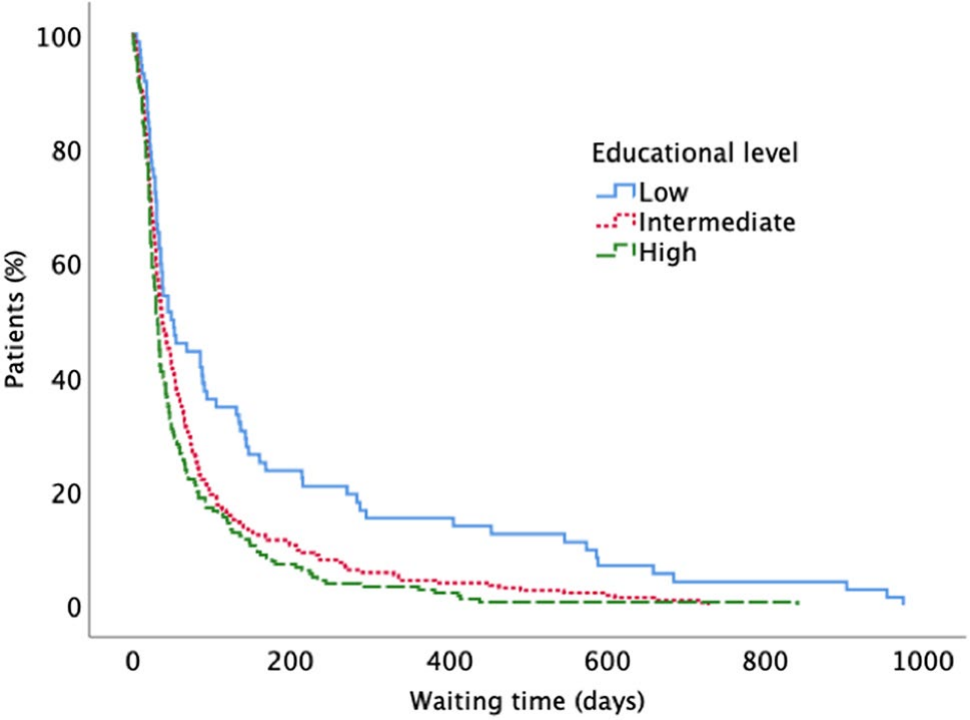
Waiting time for surgery for diffuse low grade glioma over educational groups (log-rank, p < 0.0001)

Compared to the higher education groups, patients with the lowest educational level had a worse functional status upon presentation. Furthermore, this group waited longer from imaging diagnosis to surgery than patients with higher levels of education (Fig. 1). Finally, an increased comorbidity rate was found in the lowest educational group. No significant differences between the groups were found regarding type of surgical procedure or complications.

### Sex

Similar to what was found for income and educational level, a difference in functional status at presentation was seen between women and men, with women being in a worse condition pre-operatively (Supplementary Table 1). In addition, a higher proportion of men compared to women were asymptomatic at presentation (9.9% versus 3.8%). There were no significant sex differences regarding waiting time for surgery (median 35 days for females and 36 days for males), type of surgical procedure performed, or number of complications leading to re-operation in relation to surgery.

### Survival

**Fig. 2:**
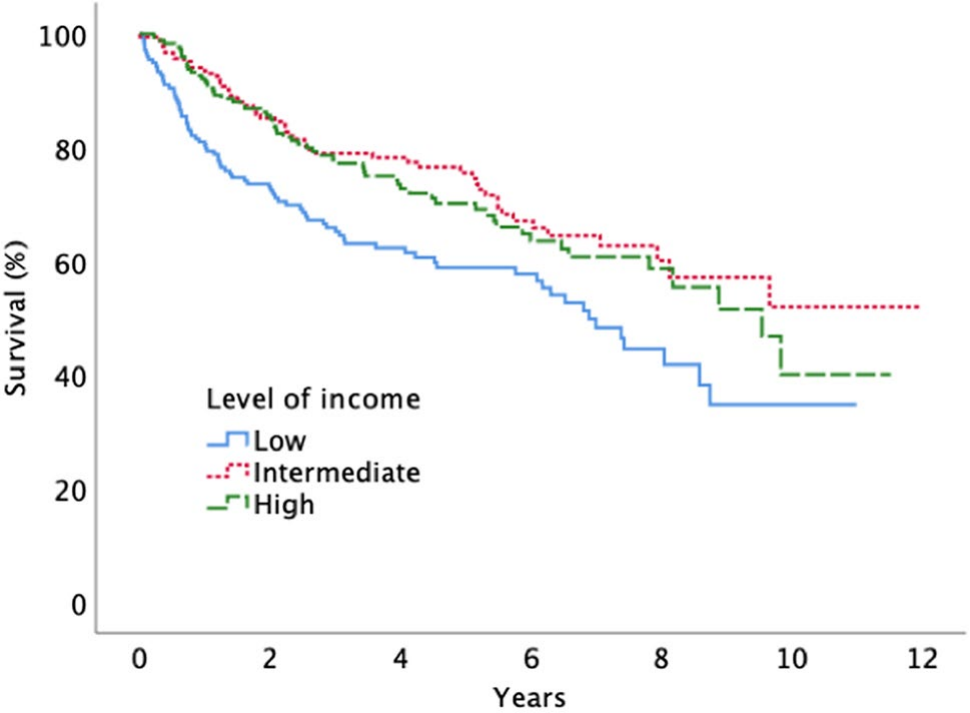
Survival after surgery for diffuse low grade glioma according to level of income (log-rank, p = 0.002)

**Fig. 3:**
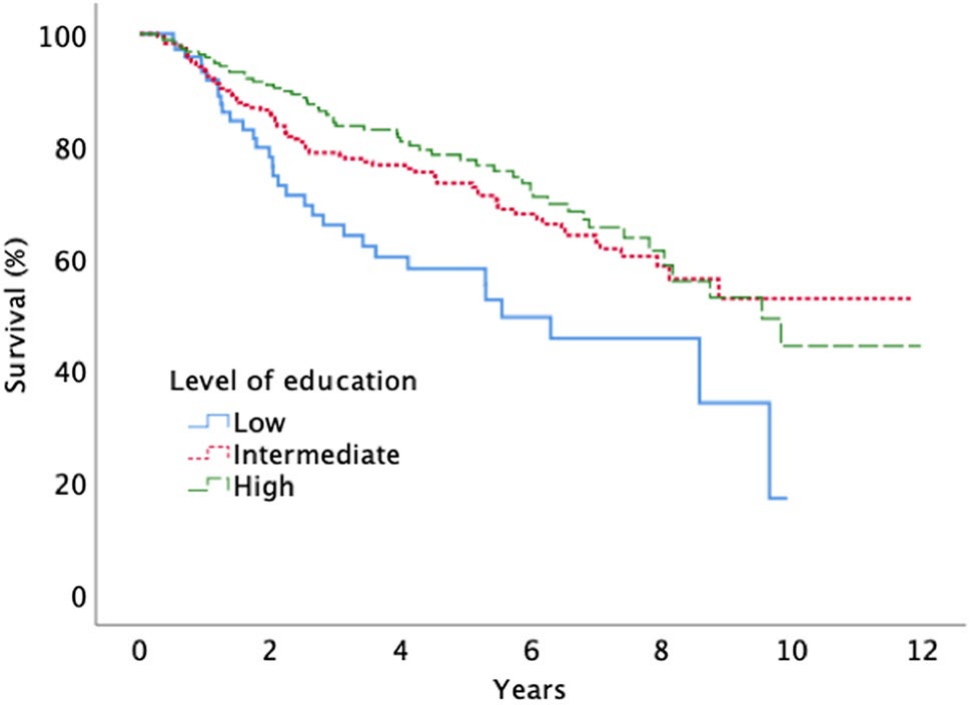
Survival after surgery for diffuse low grade glioma according to level of education (log-rank, p = 0.004)

When analyzing survival after surgery, we found that patients in the lowest income group and in the lowest educational group had shorter survival times compared to patients with higher income and education (Figs. 2, 3). No significant survival differences were seen between males and females.

### Stratification

To adjust for variables with group level differences found in earlier analyses and for factors likely to affect outcome, we made separate stratifications for age (including only patients aged 18–60), comorbidity (including only those without comorbidity) and pre-operative performance score (including only fully active patients).

When analyzing overall survival, the differences over educational levels and over income levels were no longer significant following age- and comorbidity stratification. However, for sex, a small survival difference to the benefit of females appeared in the stratum of fully active patients (log rank, p = 0.009).

Likewise stratified analyses, were made for waiting times to surgery with the addition of tumor size as a separate stratification (excluding tumors > 6 cm). All other analyses were stratified as outlined above. We decided beforehand on omitting extreme outliers (with more than three years from radiological diagnosis to surgery). The differences in waiting times were still associated with educational groups following these stratifications (age stratum log rank, p < 0.001, comorbidity stratum log rank, p = 0.001, size stratum log rank, p < 0.001, performance status stratum log rank, p = 0.03).

For groups defined by income, a significant difference in waiting times to the benefit of patients with high income level appeared in the stratum containing non-comorbid patients only (log rank, p = 0.006). No differences in waiting times between sexes were seen following the above-mentioned stratifications.

### Regional differences

To evaluate whether potential differences in waiting time and educational level between different regions in Sweden underlay the disparities seen over social groups, descriptive sub-analyses were made for the different hospital regions (Supplementary Table 2). These analyses showed interregional differences in waiting time, as well as an uneven distribution of income levels and education. However, the longer waiting times for patients with low education compared that of patients with high education were present and consistently found within each separate region.

## Discussion

In this study of neurosurgical care and outcome in a universal healthcare setting, the patterns of care for patients with diffuse LGG seemed to be influenced by their levels of education and income. Thus, patients with low education presented at a later stage (in a worse clinical condition) at time of diagnosis and waited longer for surgery compared to those with higher education. For patients with low income, a similar pattern was observed. Although less consistent, some sex imbalances were also found.

### Performance status upon presentation

We found that female patients and patients with lower level of education or income presented with worse functional status. These findings imply that patients in lower socio-economic groups may reach specialized healthcare at a later stage of their disease than those with higher SEP. Impact of socioeconomy on stage of cancer at the time of diagnosis has been observed for other types of cancer, in both market-based health care systems such as in the US [4, 8, 18], and societies with universal health coverage [8, 22, 23].

Since the nature of our study is observational, we can only speculate as to the causes of the observed differences. The so-called patient’s delay as well as doctor’s delay may be influenced by the SEP of the patient. We also observed an association between low socioeconomic status and increased comorbidity, a finding that has been described earlier [24, 25]. It is possible that an increased complexity in interpreting symptoms of individuals with a high burden of comorbidity leads to a postponed diagnosis. This hypothesis is consistent with previous findings in patients with cervical cancer, where increased comorbidity was associated with a more advanced cancer stage at diagnosis [22].

Regarding sex, the male overrepresentation in head trauma [26, 27] may lead to a higher amount of incidental findings of brain tumors on trauma-related CT scans in men. This might also explain the better performance status at presentation on the group level for males in our cohort. In accordance, we found a higher proportion of asymptomatic males compared to females in our material (p < 0.01, Supplementary Table 1). Other possible explanations might be gender differences in care-seeking behavior and unequal response from the referring physician regarding symptoms presented by males or females. In the literature, examples of ‘gender gradients’ pertaining to delayed diagnosis or delayed access to specialist care can be found in both directions [8, 28–30]. In our material, we found a slight overall survival benefit for females toward males in the subgroup of fully active patients without restrictions. It cannot be excluded that equally early diagnosing of tumors in both sexes could have the potential to further ameliorate the clinical outcome for women with LGG.

### Waiting times

We observed longer waiting times for surgery for patients in the lowest educational level group, as compared to patients with higher levels of education. Corresponding differences were found only in subgroups (non-comorbid) for income level and not at all for sex. Inequality in relation to waiting time for surgery linked to educational level is particularly disturbing in a healthcare system that has the objective of providing equal care to the population. From our stratified analyses, we noted that the observed differences in waiting times do not seem to emanate from differences in co-morbidity, age or pre-operative performance status. Also, patients with larger tumors generally are likely to get surgery more promptly but among patients with small or medium-sized tumors, those with lower education still endured longer waiting times. To exclude that the observed disparities were only a consequence of geographical differences in educational level and waiting times, we made a descriptive sub-analysis exploring each hospital region separately. The findings of disparities over socioeconomic groups were consistent and confirmed also within the separate regions.

LGG are often slow-growing tumors, and there are cases in which the benefit of surgery is limited by the risks of the procedure. The decision as to whether and when to perform surgery is normally based on individual circumstances and made in consultation with the patient, even though growing evidence for early and extensive surgery has emerged over the last decades [31]. Importantly, as can be seen in Fig. 1, the differences in waiting times related to educational level are not evident during the early phase (first 10 weeks) after diagnosis. This suggests that for urgent or evident surgical cases, the level of education of the patient does not affect waiting times, whereas social factors are likely to impact patterns of care in more complex cases.

To summarize, our results show longer waiting times for a socially underprivileged group. There are some earlier published examples of increased waiting times for surgery related to lower educational level, which in our opinion strengthen the idea that our observations are valid and not random [32–34].

### Survival

Both low education and low income were associated with shorter survival time in unadjusted analyses, but these differences disappeared in adjusted analyses. Thus, although patterns of care do seem to differ, the overall survival was unaffected by level of education and income in our healthcare setting. This lack of difference in survival is not surprising as the median difference in waiting times between for instance lower and higher education was only 19 days, and there was no difference in type of surgical procedure or surgical complications. In addition to treatment factors, survival of LGG patients is largely determined by disease-related factors and there is no reason to believe that these parameters would be unevenly distributed over socioeconomic groups. Finally, survival may be a too insensitive outcome measure. Although the lack of difference in survival is obviously reassuring, it provides us with only limited evidence for arguing that LGG patients are handled equally.

### Strengths and limitations of the present study

Our study has several limitations related to the observational design and the limited level of clinical details provided by the registers. The categorization of patients into different income and educational levels was based on the year of diagnosis and the potential ‘evolution’ of these factors over time for an individual patient has not been analyzed.

Strengths include the population-based data acquired through the standardized, consecutive, and prospective reporting to the SBTR. Most studies on socioeconomic status and health rely on area-based estimations as proxies for individual data [18, 35]. Swedish public registries normally show extraordinary coverage and data quality [36, 37], enabling us to present results on individual level from the vast majority of patients operated for LGG in Sweden in the period 2005–2015.

## Conclusions

The results of this nationwide study indicate that neurosurgical care for LGG patients is affected by socioeconomic factors also in a universal health care setting. Awareness of these aspects is a necessary first step towards successful delivery of equal care to all patients regardless of social background.

## Electronic supplementary material

Below is the link to the electronic supplementary material.

Supplementary file1 (DOCX 14 kb)

Supplementary file2 (DOCX 13 kb)
