# Supplementary material for: Socioeconomic factors affect treatment delivery for patients with low grade glioma: a Swedish population-based study
Source: J Neurooncol. 2019 Dec 27;146(2):329–37. doi: 10.1007/s11060-019-03378-7 (PMC6971149; doi:10.1007/s11060-019-03378-7)
Supplement: Supplementary file 1 — Supplementary file1 (DOCX 14 kb) [file 11060_2019_3378_MOESM1_ESM.docx]

| Supplementary Table 1. Clinico-pathological factors related to surgical treatment for diffuse, low-grade gliomas in relation to gender. | | | |
| --- | --- | --- | --- |
|  | Gender | |  |
|  | Female  n=245 (44.9) | Male  n=298 (55.1) | p-value |
| Age, mean (SD) | 46.9 (14.6) | 46.0 (15.2) | 0.48 |
| Asymptomatic, n (%) | 9 (3.8)  N=234 | 28 (9.9)  N=282 | <0.01 |
| Focal deficit, n (%) | 90 (37.5)  N=240 | 107 (37.0)  N=289 | 0.91 |
| WHO functional status, n (%)  0: fully active  1: light work possible  2: cares for self  3: limited self care  4: disabled, confined to bed | 123 (51.9)  77 (32.5)  28 (11.8)  8 (3.4)  1 (0.4)  N=237 | 176 (60.1)  58 (19.8)  44 (15.0)  9 (3.1)  6 (2.0)  N=293 | <0.01 |
| Tumor Size, n (%)  < 4 cm  4-6 cm  > 6 cm | 85 (39.5)  83 (38.6)  47 (21.9)  N=215 | 106 (40.3)  107 (40.7)  50 (19.0)  N=263 | 0.73 |
| Bilateral or multifocal tumor growth, n (%) | 26 (10.6)  N=245 | 36 (12.1 )  N=297 | 0.30 |
| Days from imaging to surgery (Q1-Q3) | 35 (21-91)  N=242 | 36 (20-83)  N=295 | 0.96 |
| Resection (not biopsy), n (%) | 168 (68.9)  N=244 | 218 (74.4)  N=293 | 0.15 |
| Postop re-operation due to complication | 14 (6.5)  N=215 | 14 (5.3)  N=262 | 0.60 |
| Histopathology:  Astrocytoma  Oligodendroglioma  Oligoastrocytoma | 114 (46.5)  100 (40.8)  31 (12.7)  N=245 | 156 (52.3)  97 (32.6)  45 (15.1)  N=298 | 0.13 |
| Number of comorbidities, n (%)  0  1  2  3 or more | 183 (74.7)  40 (16.3)  16 (6.5)  6 (2.4) | 229 (76.8)  51 (17.1)  11 (3.7)  7 (2.3) | 0.51 |

***Abbreviations:*** *WHO, World Health Organisation; postop, postoperatively.*

**The WHO/[Eastern Cooperative Oncology Group](https://en.wikipedia.org/wiki/Eastern_Cooperative_Oncology_Group) (ECOG) performance score*

***Any complication within 30 days postoperatively leading to re-operation (for example parenchymal or extracerebral hemorrhage, infection).*

*Where data are missing, the actual N is provided in individual cells.*
