# Supplementary material for: Socioeconomic factors affect treatment delivery for patients with low grade glioma: a Swedish population-based study
Source: J Neurooncol. 2019 Dec 27;146(2):329–37. doi: 10.1007/s11060-019-03378-7 (PMC6971149; doi:10.1007/s11060-019-03378-7)
Supplement: Supplementary file 2 — Supplementary file2 (DOCX 13 kb) [file 11060_2019_3378_MOESM2_ESM.docx]

| Supplementary Table 2. Median waiting time for surgery for LGG patients in lowest and highest educational group respectively and percentage of patients with high education per region. | | | |
| --- | --- | --- | --- |
| Region | Median time to surgery  – low educational level  days, (n) | Median time to surgery  – high educational level  days, (n) | Patients in region with high education |
| 1 | 35 (19) | 26 (91) | 50% |
| 2 | 118 (22) | 43 (24) | 28% |
| 3 | 46 (12) | 38 (17) | 29% |
| 4 | 144 (8) | 79,5 (34) | 37% |
| 5 | 44 (11) | 40 (17) | 30% |

*One region was not analyzed due to n=1 in lowest educational group*
